# Surface sterilization methods impact measures of internal microbial diversity in ticks

**DOI:** 10.1186/s13071-019-3517-5

**Published:** 2019-05-28

**Authors:** Florian Binetruy, Marlène Dupraz, Marie Buysse, Olivier Duron

**Affiliations:** MIVEGEC (Maladies Infectieuses et Vecteurs: Ecologie, Génétique, Evolution et Contrôle), Centre National de la Recherche Scientifique (CNRS) - Institut pour la Recherche et le Développement (IRD) - Université de Montpellier (UM), Montpellier, France

**Keywords:** *16S* rRNA, Bacterial communities, Tick microbiome, Metabarcoding, *Amblyomma*

## Abstract

**Background:**

Ticks are obligate blood feeders transmitting major pathogens worldwide. Over the past few years, considerable research efforts have focused on the diversity, distribution and impact of gut and intracellular bacterial symbionts on tick development and tick-borne pathogen transmission. The study of this internal microbiome requires the use of a sterilization method to remove external (i.e. cuticular) microbes present on the tick’s surface and to avoid any further contamination. Several sterilization methods exist, including ethanol- or bleach-based treatments that are both effective in killing microbes but with different potential effects on DNA denaturation.

**Methods:**

We examined how these different sterilization methods impact the measure of internal microbial diversity hosted by the Cayenne tick *Amblyomma cajennense* (*sensu stricto*). Bacterial barcoding investigations based on *16S* rRNA gene sequences were conducted on two batches of 50 individuals each: Ticks of the first batch were sterilized with bleach diluted at 1% and the second batch with 70% ethanol. Tick external microbiome was also determined from cuticle smearing and water samples used for tick washing.

**Results:**

Bacterial barcoding investigations showed major differences between ethanol- and bleach-treated specimens. Both methods led to the detection of major intracellular bacteria associated with *A. cajennense* (*s.s.*) but ethanol-treated ticks always harbored a higher bacterial diversity than bleach-treated ticks. Further examinations of tick gut and tick external microbiome revealed that ethanol-based surface sterilization method is inefficient to eliminate the DNA of external bacteria.

**Conclusions:**

We herein provide evidence that studies investigating the internal microbiome of ticks should consider bleach as the gold standard to efficiently remove cuticular bacterial DNA. Indeed, this method does not impact the internal bacterial diversity hosted by ticks and is thus a better method than the ethanol-based one for studying the internal microbiome.

**Electronic supplementary material:**

The online version of this article (10.1186/s13071-019-3517-5) contains supplementary material, which is available to authorized users.

## Background

Over the past 15 years, advances in genomics and microbiology have shown that metazoans commonly harbor complex microbial communities living inside and on their body, i.e. the microbiome. In arthropods, some studies have focused on cuticular (i.e. external) microbiomes leading to the description of symbiotic bacteria either protecting their hosts against superficial infections or modulating host intraspecific recognition [1–4]. However, most studies on arthropods have instead focused on the diversity and the biological importance of their internal microbiome, including the microbes living within the gut but also those living within their own cells such as maternally inherited intracellular bacteria [5–9]. It is now clear that the internal microbes of arthropods contribute to a variety of ecological and evolutionary processes, driving pivotal nutritive, reproductive and immunity functions [10–13]. Recently, some of these findings have also been discussed in the context of an eventual use of the internal microbes to limit the transmission of pathogens by blood-feeding arthropods, especially mosquitoes and ticks [14–20].

Ticks are major vectors of pathogens and especially well known for the part they play in spreading Lyme and other emerging diseases. Unlike mosquitoes, ticks feed exclusively on blood at all stages in their development and exhibit a unique internal microbiome with a diversity of non-pathogenic extracellular and intracellular bacteria [21–30]. Recent case studies have demonstrated the evolutionary and ecological importance of these internal microbes for ticks. For instance, some resident microbes of the tick gut can influence tick immune responses and then modulate their resistance to pathogens [14, 17]. Other examples include maternally inherited bacterial symbionts that are essential for ticks’ growth and survival to adulthood: the vertebrate blood ingested by ticks is limited in B vitamins, and symbionts supply these missing nutrients to them [31–33]. A deeper investigation of the biodiversity of the internal microbes of ticks is now ongoing, as shown by the increasing number of metagenomics studies using high-throughput sequencing [23, 34–36].

These case studies require the use of a sterilization protocol of the cuticles to eliminate surface microbes and exogenous DNA before investigating internal microbe diversity. Ticks are especially prone to harboring external microbes since they can be contaminated either by the skin microbiome of their vertebrate hosts during blood-feeding and, when they are “off-host,” by environmental microbes from the soil or plants [26, 37]. For these reasons, sterilization methods are commonly employed before investigating internal microbiomes, but these methods differ greatly between studies: while most studies used ethanol solutions for this purpose [14, 23, 36], a few used sodium hypochlorite (bleach) solutions instead [38, 39]. Ethanol and bleach are both effective at killing microbes but only bleach will denature DNA [40, 41]. One can thus assume that the DNA of external microbes may remain present on the tick’s cuticle after an ethanol-based sterilization. This may be an important source of contamination for internal microbial communities, and external microbes will then be misidentified as internal microbes. In addition, abundant cuticular microbes may also limit the effectiveness of next-generation microbe community profiling by masking less abundant internal microbes. However, how different sterilization methods of the tick cuticle (i.e. ethanol- *versus* bleach-based methods) impact the difference in the diversity of internal microbial communities remains entirely unknown. It is noteworthy that studies using ethanol-based sterilization methods tend to show more diverse bacterial communities [14, 17, 30, 42–46] than studies using bleach-based sterilization methods [39, 47, 48]. However, this comparison is only partly relevant since these studies did not use standardized protocols: they differ in regard to the tick species examined, but also the stages and sexes of the ticks, the sampling localities, as well as the molecular and analytical approaches. The importance of these potential biases means that a definitive comparison between ethanol- and bleach-based sterilization methods was not possible.

In this study, we thus evaluated the accuracy of the two commonly used sterilization methods for the tick cuticle: ethanol- and bleach-based. We tested the impact of these two methods on the measure of the internal microbiome of ticks, and further estimated the diversity of the cuticular microbiome through swab samples and water washes. For this purpose, 100 field adult females of the Cayenne tick *Amblyomma cajennense* (*sensu stricto*) were used as a case study. This South American tick species is restricted to the Amazonian region and its microbiome has not been investigated to date. Previous molecular investigations have, however, shown that members of the *A. cajennense* species complex commonly harbor intracellular bacteria of the *Rickettsia* and *Anaplasma* genera, which are both potential pathogens for humans and animals [49, 50]. Recent investigations have further shown that all *A. cajennense* (*s.s.*) individuals are also infected by a maternally inherited bacterium, the *Coxiella*-like endosymbiont (hereafter *Coxiella*-LE), which is assumed to be the B vitamin-providing symbiont required for tick survival [51–53].

## Methods

### Tick sampling and processing

Unfed (“questing”) adult females of *A. cajennense* (*s.s.*) were collected in October 2017 from a single locality in French Guiana (4°51′48″N, 52°20′1″’W; Piste de La Mirande). All individuals were obtained during one session, through drag-flagging on vegetation along a 100-m transect. Individuals were identified using morphological keys [54] and kept alive in sterile 50-ml Falcon tubes until their dissection or extraction of their DNA.

We randomly divided 100 *A. cajennense* (*s.s.*) female ticks into two batches of 50 individuals each. Ticks of the first batch were processed with commercial bleach diluted at 1% for 30 s and then rinsed for 1 min in three successive baths of DNA-free water. Ticks of the second batch were processed with 70% ethanol for 30 s and then rinsed for 1 min in three successive baths of DNA-free water. Directly after the baths, 25 ticks of each batch were stored in 1.5 ml of 70% ethanol prior to DNA extraction. The 25 remaining ticks from each batch were carefully dissected in a sterilized Petri dish under a stereomicroscope. Sterile scalpel blades and 21-gauge needles were used to remove cuticles, and sterile forceps were used to carefully recover the midgut that was stored in 1 ml of 70% ethanol. The rest of the tick carcass (i.e. the whole body without the gut) were also stored in 1 ml of 70% ethanol prior to DNA extraction. Between each dissection, new needles and Petri dished were used, and scalpel blades and forceps were sterilized by washing two times in sterile water and commercial bleach. Water washes of the dissection tools were further used as negative dissection controls.

To investigate and control the composition of the external microbiome, 27 additional *A. cajennense* (*s.s.*) females were subjected to a cuticle smear (ventral and dorsal faces) with sterile swabs. Furthermore, 25 other females were individually washed by vortexing for 1 min in 1.5-ml tubes full of DNA-free water, which was kept for DNA extraction. All these samples were stored at − 20 °C prior to DNA extraction.

DNA of tick samples, swabs, and water used for tick washing were extracted using a DNeasy Blood & Tissue Kit (Qiagen, Hilden, Germany) following the manufacturer’s instructions. Three negative extraction controls were included in all extraction series.

### PCR amplification and high-throughput sequencing

A 251-bp portion of the V4 variable region of the bacterial *16S* rDNA was amplified using the universal primer pair modified by Galan et al. [55] 16S-V4F (5′-GTG CCA GCM GCC GCG GTA A-3′) and 16S-V4R (5′-GGA CTA CHV GGG TWT CTA ATC C-3′). Polymerase chain reaction (PCR) was performed in a total volume of 25 µl containing 12.5 µl of Multiplex PCR Kit (Qiagen), 10 µM of each primer, 9.5 µl of DNA-free water and 1 µl of genomic DNA. PCR amplifications for each tick sample were performed in duplicate to evaluate amplification and sequencing consistency. Each PCR product was tagged with a combination of two different barcodes designed by a genomic platform (GenSeq, Montpellier University) that allows for the identification of 384 different PCR products loaded onto the same MiSeq flow cell. *16S* rDNA amplicons from external microbiome control samples were amplified, prepared and sequenced separately from tick amplicons. All PCR products were pooled and purified, and the library was constructed and sequenced by the GenSeq platform using Illumina paired-end 2 × 300-bp technology with V3 chemistry.

### *16S* rRNA data processing and taxonomic assignment

Sequence filtering criteria were applied through Illumina’s quality control procedure. All bioinformatics analyses were conducted using the pipeline FROGS [56] implemented on a Galaxy workbench [57]. First, paired-end reads were merged into contigs with the FLASH algorithm [58]; sequences not included in the size range of 200–300 bp were considered as sequencing errors and discarded. Then, chimeras were removed with the VSEARCH tool [59] and remaining sequences were clustered using SWARM [60]. Sequences with 97% similarity were clustered together and identified as an operational taxonomic unit (OTU). Each representative OTU sequence was aligned and taxonomically assigned using the Silva132 *16S* database (https://www.arb-silva.de/). Sequences that did not align to reference genes with a minimum of 80% similarity threshold were assumed to be non-bacterial *16S* rDNA and removed from further analysis. OTUs having a maximal abundance in negative controls were discarded, as described by Birer et al. [61]. False-positive OTUs were removed by filtering OTU representing less than 0.005% of the OTU total abundance [62]. Whole-tick and tick-organ sequences (guts and carcasses) were considered as different data sets and OTUs were filtered separately to maximize the probability of discarding contaminants and false-positives specific to each data set.

### Bacterial diversity and statistical analysis

To explore the difference in bacterial diversity according to the different parameters of our study, OTU sequences were used to build a phylogenetic tree using FastTree [63]. The resulting tree was used to assessed beta-diversity matrices using the generalized UniFrac (α = 0.5) index with the *GUniFrac* package in R [64]. Bar plots, non-metric multidimensional scaling (NMDS) plots, and heatmaps were generated using different FROGSTAT tools on a Galaxy workbench [56].

Amplification and sequencing repeatability were evaluated by comparing the distance matrix between PCR duplicates using permutational multivariate analysis of variance (PERMANOVA) implemented in the *vegan* package in R, and performed on the generalized UniFrac (α = 0.5) dissimilarity matrix. To determine whether the sterilization methods statistically influence the bacterial diversity of ticks, OTU sequences of whole ticks were compared as described before. To assess (i) the potential impact of the sterilization method on the internal microbiome, and (ii) the relative importance of the sterilization method *vs* tick organs in shaping the internal microbiome, a pairwise PERMANOVA was performed with all the category pairs possible between the two parameters: sterilization treatments (ethanol and bleach) and tick body parts (guts and carcasses). The pairwise PERMANOVA was conducted using the R function pairwiseAdonis [65] (https://github.com/pmartinezarbizu/pairwiseAdonis) and *P*-values were corrected for multiple comparisons using Holm’s method [66]. To control and determine the external microbiome of ticks, a pairwise PERMANOVA was performed with swabs, water used for tick washing, and ethanol-sterilized whole-tick samples. In order to compare external samples with those of ticks in a meaningful way, all known types of internal bacteria of *A. cajennense* (*s.s.*) (members of the *Coxiellaceae* and *Rickettsiaceae* bacterial families) were removed from tick samples, duplicates were merged (duplicates one + duplicates two = number of reads), and sequence data were rarefied at 2698 reads per sample (i.e. the minimal number of reads obtained here for one sample). Except for the particular case mentioned above, we performed analyses with both non-rarefied and rarefied data. All statistical tests were conducted with R version 3.5.0.

## Results

### DNA contaminants and repeatability controls

We generated *16S* rRNA gene sequences from a total of 150 samples of 100 specimens of *A. cajennense* (*s.s.*), including 50 samples from 50 whole specimens (25 ethanol-treated whole bodies and 25 bleach-treated whole bodies) and 100 samples from 50 dissected ticks (25 guts from ethanol-treated ticks, 25 guts from bleach-treated ticks, 25 carcasses of ethanol-treated ticks and 25 carcasses of bleach-treated ticks). Additional *16S* rRNA gene sequences were also generated from 27 swabs used for cuticle smearing and 25 water samples used for tick washing. The rarefaction curves confirmed that bacterial diversities were sufficiently sampled (in almost all samples). After filtration of false-positives OTUs, 4,504,538 reads distributed in 320 OTUs were obtained in the data sets of the 50 whole ticks and 6,097,046 reads distributed in 373 OTUs were obtained in the 100 tick organs samples (50 guts and 50 carcasses of ticks). We also identified 59 and 41 contaminant OTUs that had a maximum abundance in negative controls. These OTUs corresponded, respectively, to 3 and 1.7% of the total number of reads after false-positive filtration. The two most abundant contaminants were affiliated to chloroplasts and *Streptococcus* bacteria.

No difference in bacterial composition and diversity was observed between PCR duplicates (Additional file 1: Figure S1; PERMANOVA whole ticks, *R*^2^ = 0.006, *P* = 0.59; tick organs, *R*^2^ = 0.004, *P* = 0.51). PCR duplicates of the same sample were thus pooled for further analyses. The vitamin B-providing symbiont *Coxiella*-LE was consistently observed in all *A. cajennense* (*s.s.*) samples, while the putative pathogen *Rickettsia* was only observed in some of them as further detailed below. These two intracellular bacteria were not detected from swab and water samples.

### Comparison of surface sterilization methods on bacterial diversity from tick whole bodies

**Fig. 1:**
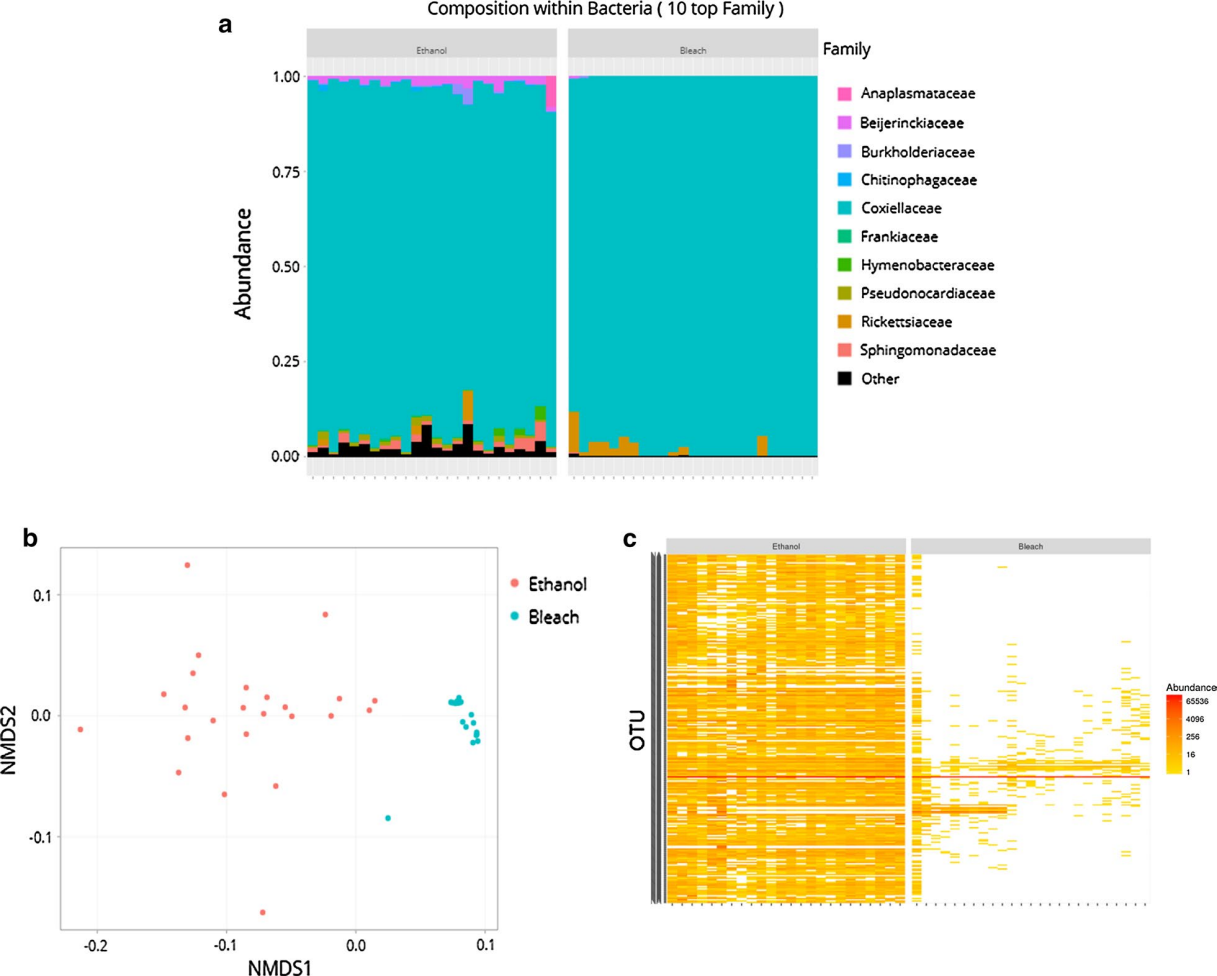
Effect of the sterilization treatment on bacterial diversity of whole ticks. **a** Bar plots of the relative abundance of the 10 most abundant bacterial families in each sample. Each bar represents a sample; ethanol samples on the left and bleach samples on the right. **b** NMDS plot of generalized Unifrac (α = 0.5) distances between treatments; blue dots correspond to bleach samples, red dots to ethanol samples. **c** Heatmap of the diversity and abundance of OTUs between bleach (on the right) and ethanol (on the left) samples with the different samples on the X axis and OTUs on the Y axis. The most common OTU, *Coxiella*-LE of the family *Coxiellaceae*, is shared by all samples and is delineated by the ‘red line’ across the heatmap

We first compared the results of the 25 ethanol-treated whole bodies and of the 25 bleach-treated whole bodies of *A. cajennense* (*s.s.*) females (Fig. 1a–c). Ethanol-treated ticks produced more than twice as many reads (mean ± SE: 58,218 ± 17,817 reads) as bleach-treated ticks (26,877 ± 20,477; Wilcoxon two-tailed test, *W* = 363, *P* = 9.9e−10; Additional file 2: Table S1). Each ethanol- and bleach-treated whole-body sample was highly dominated by one taxon with more than 90% (from 93% to 100%) of the reads assigned to a member of the family *Coxiellaceae*, *Coxiella*-LE (Fig. 1a). The second most abundant taxon was a member of the family *Rickettsiaceae*, *Rickettsia*, although it was heterogeneously distributed within the samples and detected in four and ten ethanol- and bleach-treated samples, respectively (Fig. 1a). However, bacterial diversity patterns clearly differed between the two different sterilization methods. There was significant variation in the bacterial diversity between ethanol-treated samples while only a few variations were apparent between the bleach-treated samples, with most observations clustering together on the NMDS plot (Fig. 1b). This difference was also consistent in the heatmap: many OTUs, most showing a low-to-medium abundance such as *Sphingomonadaceae* or *Beijerinckiaceae* were widely present in ethanol-treated samples while only very few OTUs were present in bleach-treated samples (Fig. 1c). PERMANOVA analysis further confirmed that the sterilization method explains most of the bacterial diversity variation between samples (*R*^2^ = 0.57, *P* = 0.001). Overall, this makes clear that bleach-treated whole-body samples exhibited a lower bacterial diversity than ethanol-treated samples.

### Characterization of internal microbial diversity

**Fig. 2:**
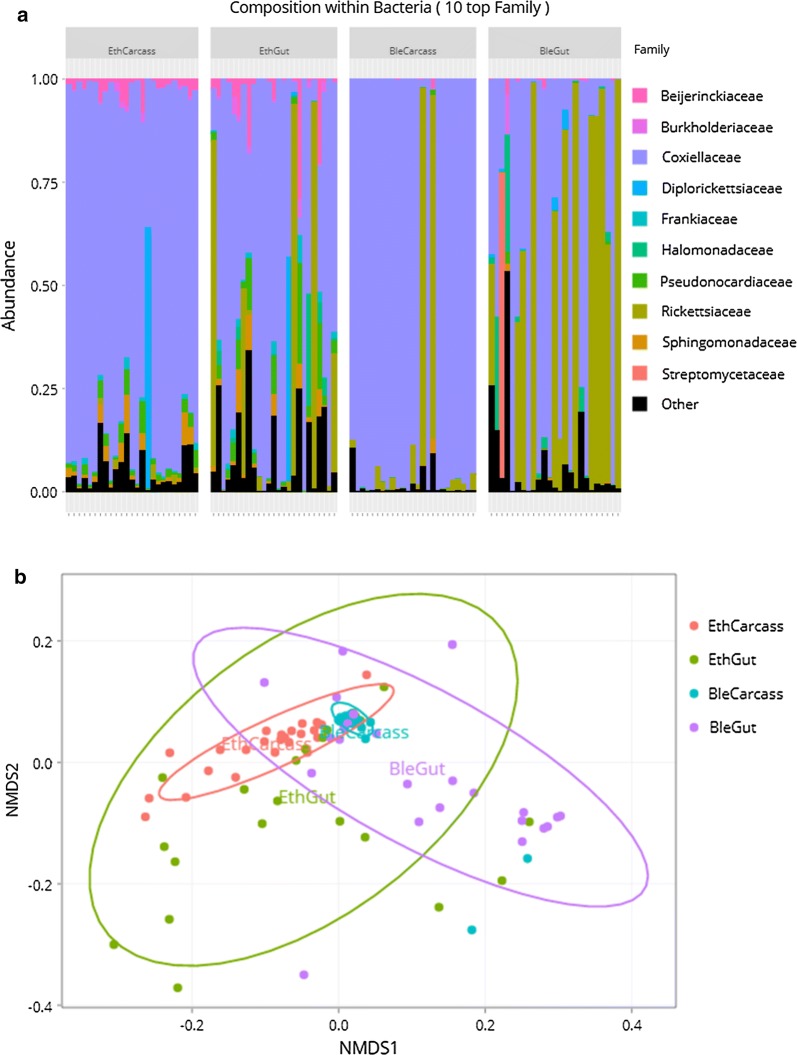
Effect of the sterilization treatment on the internal microbiome of ticks. **a** Bar plots of the relative abundance of the 10 most abundant bacterial families in each sample regrouped by sample categories; EthCarcass corresponds to ethanol-treated carcass ticks, EthGut to ethanol midguts, BleCarcass to bleached carcass ticks, and BleGut to bleached tick midguts. **b** NMDS plot of generalized Unifrac (α = 0.5) distances between all categories with ellipses encompassing normal confidence range for each category

The bacterial diversity observed suggests that bleach is more effective for removing external microbes than ethanol. However, one may also assume that bleach was internalized during the sterilization process and then denatured the DNA of internal microbes. This may explain why bleach-treated samples exhibited a lower bacterial diversity than ethanol-treated samples. To examine this possibility, we further assessed the bacterial diversity present in the guts of ethanol- and bleach-treated *A. cajennense* (*s.s.*) females. To this aim, we used 25 guts from ethanol-treated ticks, 25 guts from bleach-treated ticks, 25 carcasses of ethanol-treated ticks and 25 carcasses of bleach-treated ticks (Fig. 2a, b; Additional file 3: Table S2). Guts consistently harbored a higher diversity than carcasses in the bleach- (pairwise PERMANOVA, *R*^2^ = 0.327, adjusted *P* for multiple comparisons = 0.0006) and ethanol-treated specimens (pairwise PERMANOVA, *R*^2^ = 0.09, adjusted *P* for multiple comparisons = 0.0308; Fig. 2). Carcasses of bleach-treated ticks showed a lower bacterial diversity than carcasses of ethanol-treated ticks (pairwise PERMANOVA, *R*^2^ = 0.368, adjusted *P* for multiple comparisons = 0.0006; Fig. 2a, b), thereby corroborating our previous observations on tick whole bodies. While there was a significant structural change between the gut communities between guts from ethanol- and bleach-treated ticks (*F* = 40, *P* < 0.0001), no significant difference in bacterial diversity was observed (pairwise PERMANOVA, *R*^2^ = 0.03, adjusted *P* for multiple comparisons = 0.18; Fig. 2a, b), showing that surface sterilization protocols impact the gut bacterial diversity in the same way.

### Detection of external microbes

**Fig. 3:**
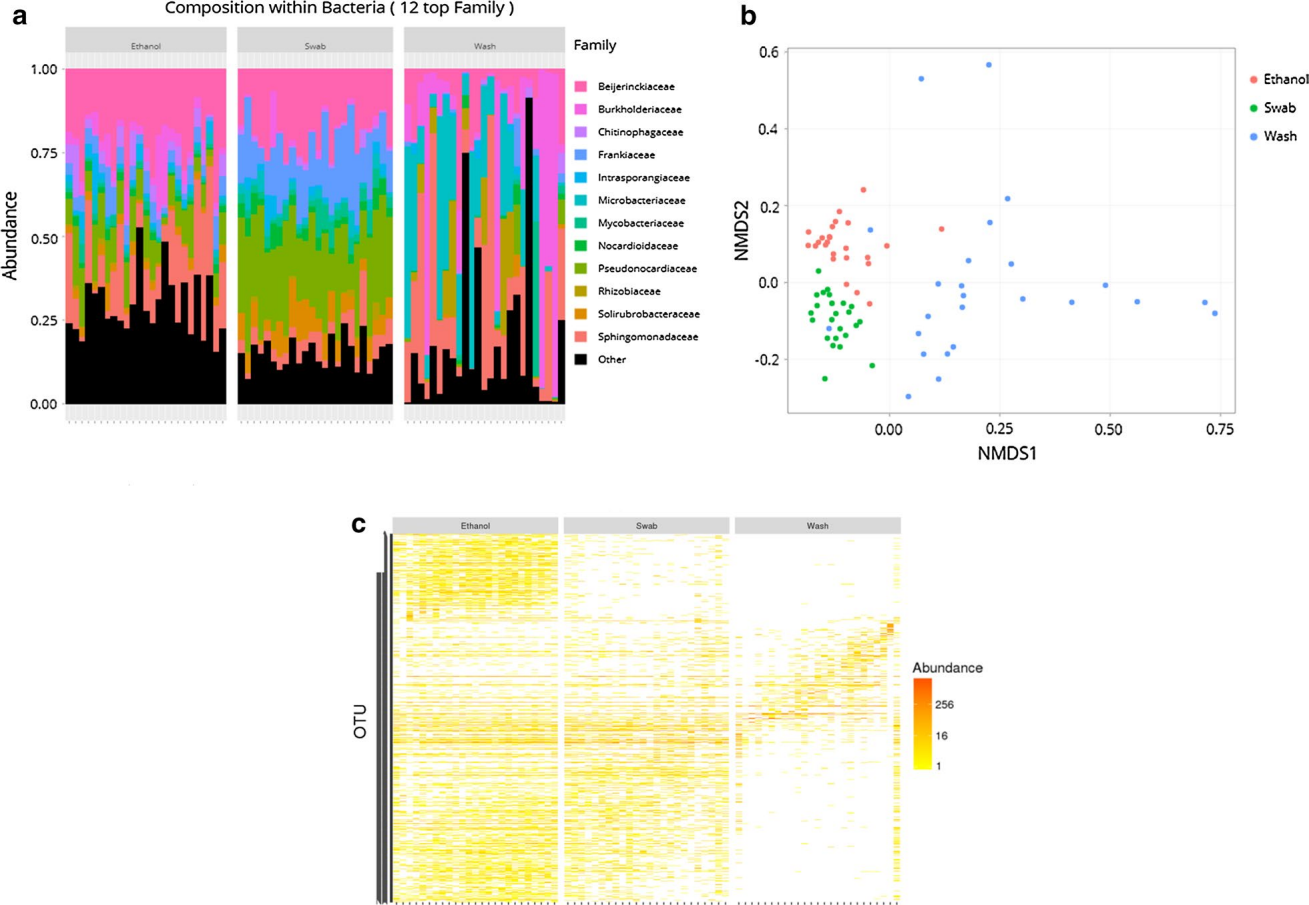
Comparison of microbial communities between cuticle smears, external washes, and ethanol-treated whole bodies. **a** Bar plots of the relative abundance of the 12 most abundant families in each sample regrouped by samples category (ethanol for whole bodies, swab for cuticle smears, and wash). **b** NMDS plot of generalized Unifrac (α = 0.5) distances between samples categories. **c** Heatmap of the diversity and abundance of OTUs between samples categories

To characterize the external microbes of *A. cajennense* (*s.s.*) females, we characterized the microbial communities of 27 cuticular smears and 25 water samples used for external cleaning (Fig. 3a–c). We obtained 1,433,868 reads leading to the identification of 859 OTUs after filtration of false-positive OTUs. We further compared this diversity with the one observed in the 25 ethanol-treated whole bodies described earlier. All known intracellular symbionts (e.g. *Coxiella*, *Rickettsia*) of ticks were discarded from the whole-tick data, resulting in only ethanol-treated ticks being used for this analysis since intracellular symbionts represented over 98% of the microbial community in bleached ticks (Additional file 4: Table S3). The bacteria community retrieved in external washes was clearly different to the two others observed in swabs and ethanol-treated whole bodies (Fig. 3a–c). Indeed, the taxa composition was far more heterogeneous across samples of the external wash categories than those of the other two (Fig. 3a). The microbial diversity of the wash samples was dominated by *Burkholderiaceae*, *Microbacteriaceae*, *Sphingomonadaceae* and *Beijerinckiaceae* (Fig. 3a). These taxa were also present in ethanol and swab samples, but only the *Beijerinckiaceae* and *Sphingomonadaceae* families were additionally highly abundant (Fig. 3a). However, in contrast to ethanol, swab samples were also dominated by *Frankiaceae* and *Pseudonocardiaceae* families (Fig. 3a). These results are clearly illustrated in the NMDS plot, where wash samples are widely distributed reflecting the heterogeneity of bacteria diversity across samples (Fig. 3b). By contrast, the ethanol and swab data set are clustered in the left of the X axis and differ only by small amounts in the Y axis (Fig. 3b). A similar pattern is observed in the heatmap (Fig. 3c): wash samples differ greatly from ethanol samples and swab samples, which differ by a cluster of OTUs only present in the ethanol samples. The three pairs tested with the pairwise PERMANOVA show a significant difference in all categories: ethanol *vs* swab (*R*^2^ = 0.31, adjusted *P* for multiple comparisons = 3e−04), ethanol *vs* wash (*R*^2^ = 0.50, adjusted *P* for multiple comparisons = 3e−04) and swab *vs* wash (*R*^2^ = 0.56, adjusted *P* for multiple comparisons = 3e−04).

## Discussion

In the present study, we evaluated the effect of the two most common methods, one based on ethanol and the other on bleach, used to remove microbe contaminants present on tick cuticles. Although most previous studies on the internal microbiome of ticks commonly used an ethanol-based method [14, 17, 30, 42–46], we observed systematic differences between ethanol- and bleach-treated specimens. Both methods led to the detection of the B vitamin-providing symbiont *Coxiella*-LE in all *A. cajennense* (*s.s.*) samples, and of a putative pathogen *Rickettsia* in some of them, as expected from previous studies [50–53]. However, despite these obvious similarities, ethanol-treated ticks consistently harbored a higher bacterial diversity than bleach-treated ticks. In this context, further observations are particularly relevant: (i) there was no difference in bacterial diversity between ethanol- and bleach-treated ticks, showing that surface sterilization methods impact the internal microbiome in the same way; (ii) the bacterial diversity of cuticle smears was very similar to the one found in ethanol-treated ticks, but not to the one of bleach-treated ticks. Most of the taxa retrieved in the cuticle smears and ethanol-treated ticks are known to be environmental bacteria associated with soil and plants (i.e. *Beijerinckiaceae* [67]) or to be cuticular symbionts of arthropods (i.e. *Pseudonocardiaceae* [68]). Overall, these findings prove that the ethanol-based surface sterilization method is not efficient to eliminate DNA of external bacteria and could lead to DNA contamination from the cuticle during tick dissection. By contrast, the bleach-based surface sterilization method can denature the DNA of external bacteria and is thus a better practice for studies aiming to characterize the internal microbiome of ticks.

The low bacterial diversity observed here in bleach-treated *A. cajennense* (*s.s.*) ticks, along with previous studies using bleach-treated specimens of other tick species [39, 47, 48], supports the recent finding that ticks harbor a rather simple internal microbiome dominated by maternally inherited symbionts [69, 70]. Indeed, *Coxiella*-LE alone represents the quasi-totality of the internal microbiome of *A. cajennense* (*s.s.*) females. This suggests that only few other internal bacteria are present but, alternatively, one can also assume that the abundance of *Coxiella*-LE *16S* rDNA reads masks the presence of less abundant bacteria. The rarefaction curves of our samples and the conclusions of previous studies [39, 69, 70] indicate that such a low bacterial diversity, highly dominated by intracellular symbionts, is a biological reality in ticks. On the other hand, in a study of the Australian tick *Ixodes holocyclus* [38], the authors successfully eliminated a maternally inherited symbiont, *Midichloria*, using blocking primers and showed a significant increase of bacterial diversity in *Midichloria-*free samples. However, all these studies confirm that maternally inherited endosymbionts are the major bacterial partner of ticks.

## Conclusions

In conclusion, we herein provide evidence that studies investigating the internal microbiome of ticks should consider commercial bleach as the gold standard to efficiently remove cuticular bacterial DNA. We used a standardized 30 second bleach treatment, sufficient to remove external microbes, although it is obvious that a shorter or longer time may affect the result. As such, prior studies investigating the microbiome without bleach surface sterilization should be reconsidered in light of our results. Moreover, this study contributes evidence supporting the new paradigm that a highly diversified and complex gut microbiome is not shared by all arthropods [69–72]. Interestingly, this lack of complex gut microbiome seems to be shared by arthropods specialized in a restricted diet, such as blood or plant sap: these arthropods commonly harbor one or two types of maternally inherited symbionts able to satisfy most of the nutritional requirements of their hosts [6, 8, 13, 73–76]. Such maternally inherited symbionts may render facultative the presence of other internal microbes, leading to an internal microbiome of low complexity. This suggests a role of these nutritive symbionts in shaping the gut microbiome of arthropods specialized in a restricted diet.

## Additional files

**Additional file 1: Figure S1.** Effect of PCR duplicates on bacterial diversity. Nonmetric multidimensional scaling (NMDS) plot of generalized Unifrac (α = 0.5) distances between PCR duplicates of samples: **a** Whole ticks, **b** tick organs. Blue dots correspond to first duplicates (D1), red dots to second (D2). **c** Heatmap plot showing abundance of OTUs across whole-tick samples and d abundance of OTUs across tick-organ samples. X and Y axes show the different samples and OTUs, respectively. D1 heatmaps correspond to first duplicates while D2 corresponds to the second ones.

**Additional file 2: Table S1.** OTU abundance without contaminants retrieved in whole-tick samples.

**Additional file 3: Table S2.** OTU abundance without contaminants retrieved in tick-organ samples (guts and carcasses).

**Additional file 4: Table S3.** OTU abundance without contaminants and intracellular endosymbiont retrieved in cuticle smears, wash samples, and ethanol-treated whole-tick samples.

## Data Availability

The datasets supporting the conclusions of this article are included within the additional files and raw sequencing data are available in the GenBank database under the Accession Number PRJNA530927.

## Abbreviations

LE: like endosymbiont
OTU: operational taxonomic unit
NMDS: non-metric multidimensional scaling
PERMANOVA: permutational multivariate analysis of variance
