# Supplementary figures and images for: Surface sterilization methods impact measures of internal microbial diversity in ticks
Source: Parasit Vectors. 2019 May 28;12:268. doi: 10.1186/s13071-019-3517-5 (PMC6537145; doi:10.1186/s13071-019-3517-5)

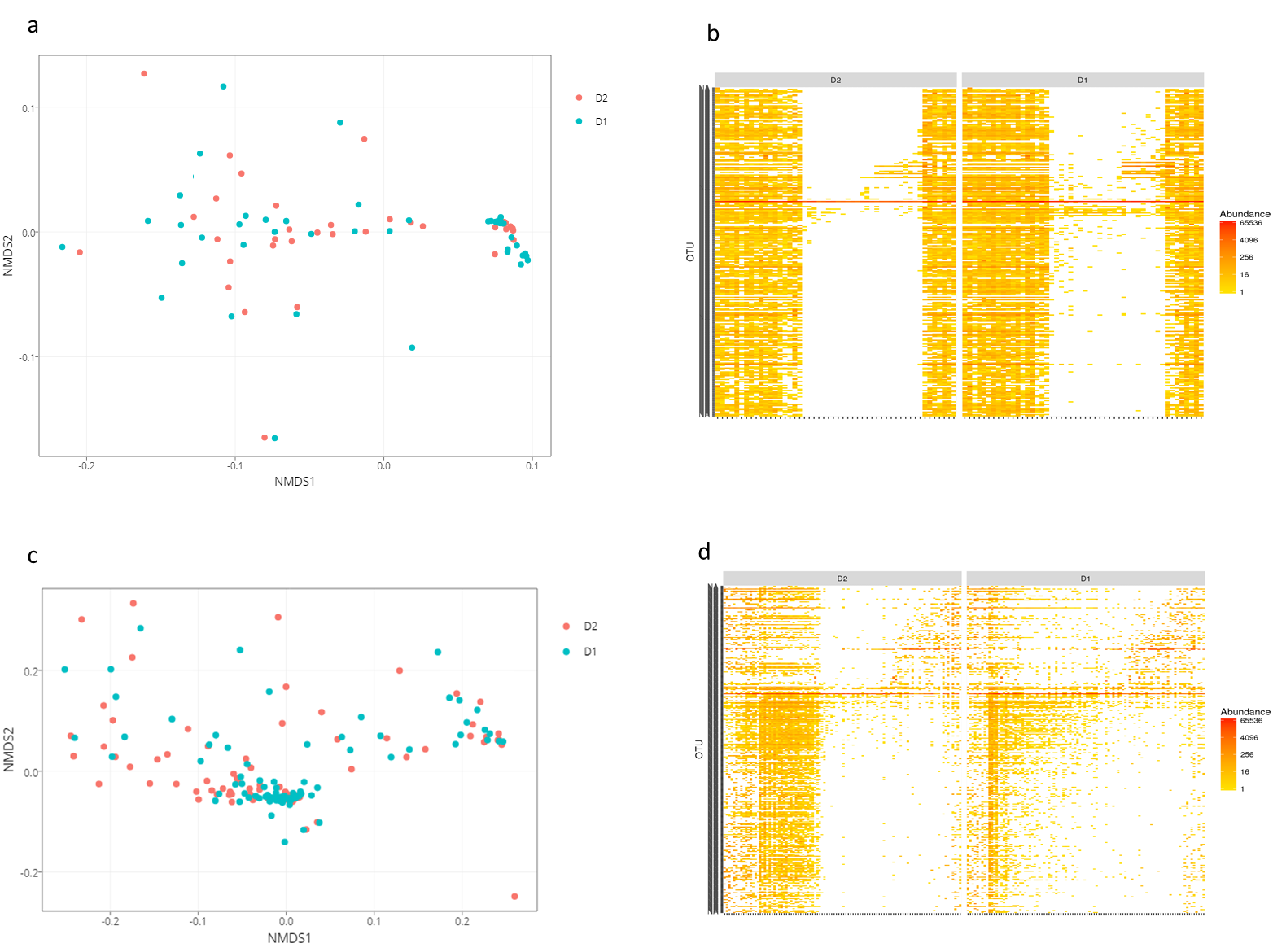

Supplement: Supplementary file 1 — Additional file 1: Figure S1. Effect of PCR duplicates on bacterial diversity. Nonmetric multidimensional scaling (NMDS) plot of generalized Unifrac (α = 0.5) distances between PCR duplicates of samples: a Whole ticks, b tick organs. Blue dots correspond to first duplicates (D1), red dots to second (D2). c Heatmap plot showing abundance of OTUs across whole-tick samples and d abundance of OTUs across tick-organ samples. X and Y axes show the different samples and OTUs, respectively. D1 heatmaps correspond to first duplicates while D2 corresponds to the second ones. [file 13071_2019_3517_MOESM1_ESM.png]
